# Bethlem Hospital

**Published:** 1852-07-01

**Authors:** 


### BETHLEM HOSPITAL.

Most of our readers are, in all probability, aware that a successful attempt was made when the Earl of Shaftesbury's bill for the regulation of asylums was passing through the House of Lords, to exempt Bethlem Hospital from the official visitation and control of the commissioners in lunacy. These gentlemen have, from time to time, had various statements laid before them as to the alleged gross mismanagement of this hospital, and desirous of ascertaining the accuracy of the reports in circulation, the commissioners applied to the Secretary of State for the Home Department, for permission to officially examine into the condition of the institution and to ascertain the treatment to which the patients were subjected. Armed with the authority of government, the commissioners instituted a rigid inquiry into the state of Bethlem Hospital; medical officers, attendants, governors, and patients were examined upon oath at the hospital, and before the Board of Commissioners. Discoveries of a most painful character are said to have been made both in regard to the general mismanagement of the asylum, as well as to the cruel treatment of the patients. The official report of the commissioners to the Home Office is represented to be anything but flattering to the vanity of those officially connected with the hospital. Dr. Munro and Sir A. Morison protest against any report having been made without being afforded an opportunity of answering the charges made against their conduct as the physicians of the asylum. These gentlemen have applied to the commissioners, and to Mr. Secretary Walpole, for a copy of the evidence upon which the adverse report of the commissioners is based, but without success. The commissioners have no objection to a publication of the evidence and report—the *hitch* exists at the Home Office. It now appears that Mr. Walpole is willing to lay a copy of the report, evidence, correspondence, &c., upon the table of the House of Commons, as soon as the committee, having the general management of Bethlem, send in their reply to the charges of negligence and inattention made against them by the witnesses examined by the commissioners. We hope yet to be in possession of a copy of the report of the commissioners before we go to press; if so, we intend publishing it *in extenso*. The evidence, we understand, is voluminous; therefore it will be impossible to print in this number of our journal an analysis of it. In consequence of the inquiry of the commissioners, and, we presume, the suggestions embodied in their report, the governors of the hospital have resolved to materially alter its medical organization, by appointing a resident officer of a superior grade, who is to have for the future the exclusive management of the hospital and treatment of the patients, with a salary of £700 per annum, exclusive of a furnished residence, coals, &c. The appointment is said to be (inclusive of pupils, fees, &c.) equivalent to £1000 a year. We hail with feelings of unqualified and unbounded satisfaction this recognition of the pecuniary value of medical services. The salary offered is a good one, and may tempt a few men indifferent as to private practice to become candidates for the office. We have before us a programme of the duties which will devolve upon the new resident physician and medical superintendent. In the first place, he is not professionally to attend any private patient, or to be connected directly or indirectly with any other establishment for the reception of lunatics. During his term of office he is never to leave the hospital without placing some other competent medical officer in his place, such substitute having the approbation of the "president and treasurer." This, we think, might have been dispensed with. Surely, if a good, trustworthy, and efficient man be selected, it would have placed him in a more comfortable position, to have freed him from the necessity of being obliged, whenever he wished to leave the hospital, to apply to the "president and treasurer" for their approval of the party selected to act for him during his temporary absence.

Again, the medical superintendent's position is rendered more dependent by the fact that he is never to be absent from the hospital *for one night,* "without the written assent of the president or treasurer," but an absence of one month in every year is to be permitted, "provided that arrangements are made satisfactorily to the same authorities." The medical officer is to be under the control of the president, treasurer, committee, and the Bethlem sub-committee; but in the hospital he is to reign supreme, having the exclusive management of all the other officers, attendants, and servants, and the medical and moral treatment of the patients. What are the duties of the resident superintendent? He is to classify the patients, regulate the diet tables from time to time, examine the provisions furnished for the use of the patients; to examine every patient upon admission, and, if requisite, on their discharge,—to have proper entries made in the books; to make daily visits to all parts of the hospital, &c. He is to visit every patient under restraint or seclusion, to exercise a general supervision over all the attendants and servants, to act as physician to the other resident medical officers, and also to all the inmates of the house of occupation—to see that prescriptions *are dispensed and administered*—to investigate all complaints made by patients, attendants, and servants—to take care that the prescription books, medical journals, and case books be regularly kept. The minute particulars as to each patient are to be entered into the case book, including the previous history of the case, with an accurate description of the external appearance of each patient—the character of the eyes, the expression of the countenance, and the peculiarities of physical formation, with a minute description of the early and more advanced symptoms of the attack.

The resident superintendent is to direct the *post-mortem* examinations, communicate with the relatives of patients dangerously ill, and "to *remain* with them, as far as may be practicable, during the continuance of such illness;" he is to admit medical pupils to the hospital; make regulations for their instruction; to give each term a course of lectures, to be illustrated by cases under his care at the time; and to present to the General Court of Governors in every year an annual report, &c. &c.

In sober seriousness, we ask where is the man to be found, irrespectively of his mental qualifications, with a sufficient *physique* to enable him to perform *one-fourth* of the duties that are to devolve upon the resident medical officer of Bethlem Hospital? Unfortunately for this great institution, giants either in body or intellect do not exist in these degenerate days; yet one would suppose that in dotting down the details of the medical officer's duty, the governors of Bethlem had some modern Goliath, or Admirable Crichton, in view, otherwise how could they, for one moment, have magined that any one man would be able to approach the standard which the governors of Bethlem evidently had in contemplation whilst compiling the document before us! If, instead of £700, they were to offer £7000 per annum to their medical officer, he never could perform a tithe of the duties expected of him with any degree of satisfaction to a conscientious mind, or advantage to the patients who would be under his care. If they were to appoint one officer to keep a record of the cases, to make out the reports, to attend the meetings of the committee; another to compound the medicine, and to see it administered; a third to attend to and visit the sick, see and correspond with the relatives; and *two* to visit and prescribe for the patients, to make the *post-mortem* investigations, and to lecture,—there then would exist an efficient medical staff, and we should have reasonable expectations of witnessing Bethlem Hospital take a position with *Charenton* and the *Bicetre*. We feel positively assured that there is ample work at Bethlem Hospital for four resident medical officers. How is it possible that, with the immense amount of responsibility and work devolving upon the resident medical officer, the governors can expect that he will have time to prepare a course of lectures, in accordance with the most recent discoveries in the pathology and therapeutics of insanity, likely to reflect credit upon himself, and to elevate in public estimation Bethlem Hospital as a scientific school of medicine? We do not for a moment doubt, with a few exceptions, the utility of the regulations laid down for the guidance of the medical officer, but we maintain that no *one human being* can be found competent to discharge them at all efficiently.

